# Fine-scale abundance of rocky shore macroalgae species with distribution limits in NW Iberia in 2020/2021

**DOI:** 10.3897/BDJ.10.e80798

**Published:** 2022-04-04

**Authors:** Joana Pereira, Catia Monteiro, Rui Seabra, Fernando P. Lima

**Affiliations:** 1 CIBIO/InBIO, Universidade do Porto, Porto, Portugal CIBIO/InBIO, Universidade do Porto Porto Portugal

**Keywords:** north-western Iberia, macroalgae, intertidal, distribution, rocky shores

## Abstract

**Background:**

Climate change has been increasing at an unprecedented rate in the last decades. Global warming has been causing a variety of impacts in marine ecosystems, including shifts in the geographical ranges of species. The north-western Iberian Peninsula coast is particularly interesting to study distribution shifts as it features a strong latitude thermal gradient, establishing a biogeographical transitional region where several cold- and warm-adapted species have their equatorward or poleward distributions. In the early 2000s, it appeared that, while warm-water species were already responding to warming, cold-water species did not display a coherent response. It is now necessary to gather up-to-date data on the distribution of the same group of species to understand if current patterns of change confirm or deny those observed back then, which may give us important clues about the mechanisms setting species limits in the area.

**New information:**

This study provides a fine-scale description of the occurrence of intertidal macroalgae species in the rocky shores of the north-western Iberian coast. Specifically, the spatial distribution and semi-quantitative abundance of 34 native and invasive species were assessed at 70 wave-exposed locations. This included 19 species of cold-water affinity, 10 species of warm-water affinity and five neutral species. When contrasted with historical observations, these new data can be used to quantify and map biodiversity change in the region, as well as help understanding the mechanisms constraining species distributions.

## Introduction

Climate has been changing at unprecedented rates (IPCC 2021). Amongst the most pervasive effects of climate change have been shifts in the geographical distribution of species (Parmesan and Yohe 2003), generally tracking isotherms towards the poles (Lenoir et al. 2020).

Intertidal species are considered sensitive indicators of climate change (Southward et al. 1995), as they are particularly vulnerable to environmental extremes, including high temperatures (Sorte et al. 2016, Zamir et al. 2018). The North-western Iberian Peninsula coast features a strong latitude thermal gradient, establishing a biogeographical transitional region where several cold- and warm-adapted species have their equatorward or poleward distribution limits (Fischer-Piette 1959, Fischer-Piette and Gaillard 1959, Fischer-Piette 1963, Ardré 1970, Lima et al. 2007, Araújo et al. 2009) and where shifts in those limits have been described since the 1950s (Fischer-Piette and Forest 1951, Fischer-Piette 1957, Fischer-Piette and Prenant 1957, Fischer-Piette 1960, Berke et al. 2010, Wethey et al. 2011, Sousa et al. 2012, Rubal et al. 2013). In the summer, the latitudinal thermal gradient is further intensified by the cooling effect of the Canary upwelling system, stronger in the northern portion of the coast. Recent studies suggest that this cooling may be, at least partially, buffering coastal ecosystems from decades of global warming (Seabra et al. 2019), but the extent to which this is actually happening has not yet been verified in the field.

Anticipating a prevalence of distributional shifts towards the north, in the early 2000s Lima and colleagues conducted several surveys in the area (Lima et al. 2007, Pereira et al. 2021c), finding that, while all warm-water species that were changing were expanding their range northwards, cold-water species showed no particular shifting trend as the number that retreated north or expanded south was the same. It seemed, at the time, that warm-water species were already responding to warming, but the same could not be said about cold-water species (Lima et al. 2007). Although it is likely that communities kept changing since those observations in the early 2000s (Harley et al. 2012), detailed up-to-date information is not available. Budgetary constraints and logistical limitations inherent to field surveys mean that data are often outdated or poor in details (Casado-Amezúa et al. 2019). Most recent surveys in the area lack the necessary taxonomic coverage (Araújo et al. 2011, Assis et al. 2017), geographical range (Piñeiro-Corbeira et al. 2016) or spatial resolution (Meneghesso 2020) to either pinpoint the exact distribution limits of those species or to evaluate changes in abundance towards those limits.

It is essential to gather fresh data on the distribution of the same group of species studied more than one decade ago to understand if current patterns of change confirm or deny those observed back then. Determining the generalisation of those observations is important, as they may give us clues on the mechanisms constraining species distributions (which may even differ between warm and cool range edges, see Paquette and Hargreaves 2021). In an effort to reduce this knowledge gap, this study aimed at re-assessing the abundance and the distribution limits of 34 native and invasive species of rocky shore macroalgae species with high spatial accuracy, at 70 wave-exposed locations along the north-western coast of the Iberian Peninsula.

## Sampling methods

### Study extent

**Figure 1.**
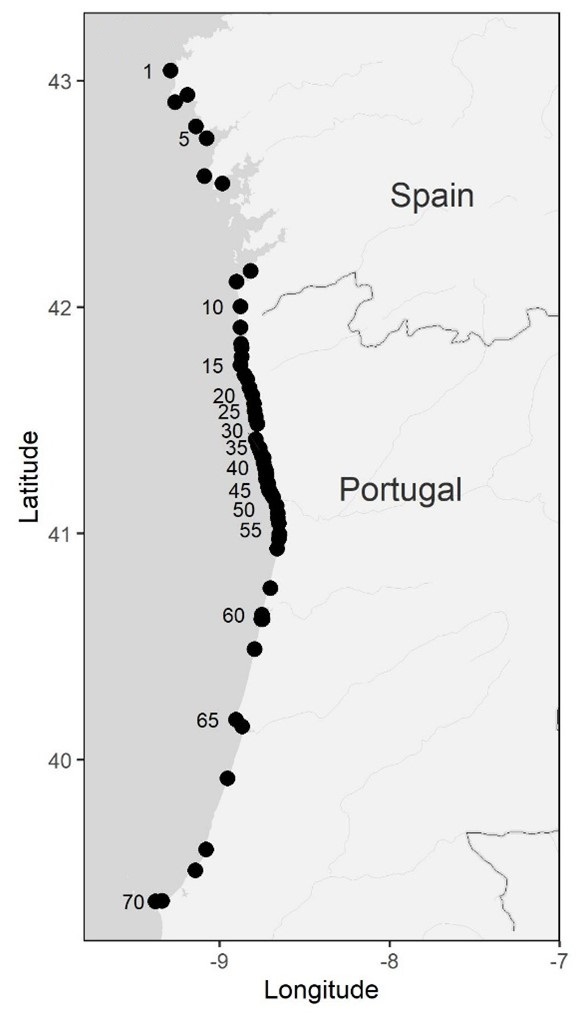
Study locations along the north-western Iberia coast visited in the years of 2020 and 2021. Location details and sampling dates can be found in Table 1 and Table 2.

**Figure 2.**
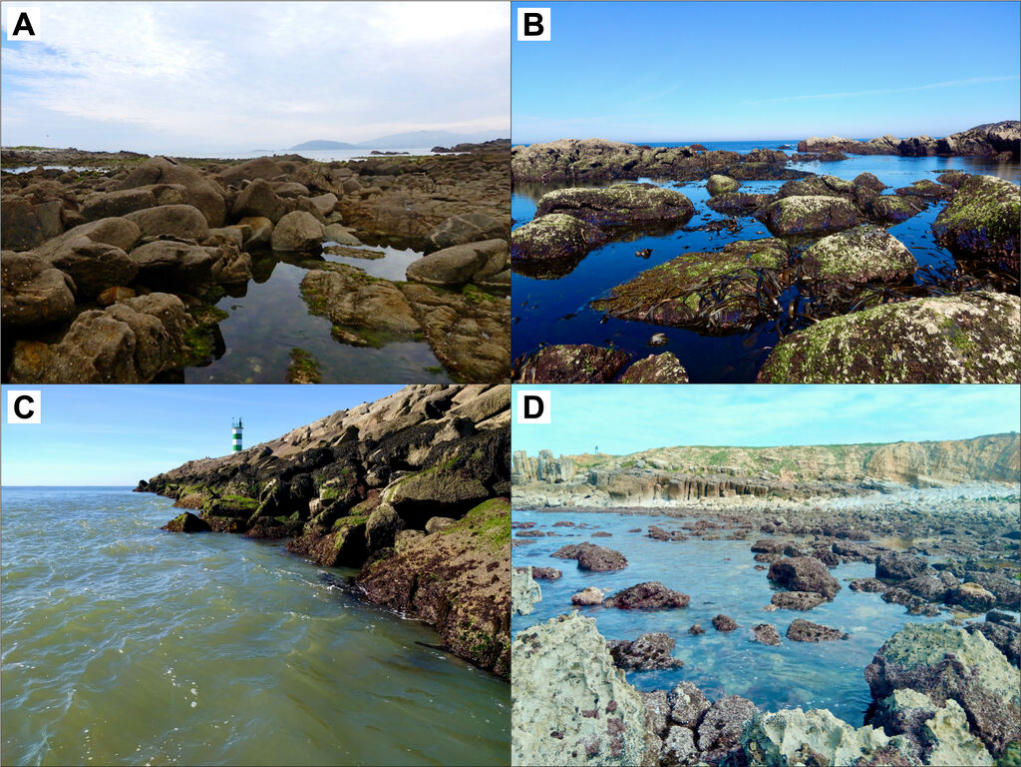
Examples of shores surveyed in the present study. A - Oia, in Galicia, B – Moledo, in northern Portugal and C – Cabedelo (breakwater), an artificial site in northern Portugal. D – Papôa, in central Portugal.

Data were collected between autumn 2020 and summer 2021. Sampling was carried out at 70 wave-exposed rocky shore locations along the north-western Iberian coast, covering three major rocky stretches of shoreline in the regions of Galicia, northern Portugal and central Portugal (Table 1, Table 2, Fig. 1, Fig. 2). In Galicia, we sampled the locations of Cabo Touriñán, Corveiro, Quenxe, Ximprón, Punta Outeiriño, Corrubedo, O Touro, Prado, Faro Vello de Silleiro, Oia and Fedorento. In northern Portugal, we surveyed the locations of Moledo, Vila Praia de Âncora, Afife, Montedor, Forte da Vigia, Praia Norte, Cabedelo (breakwater), Amorosa, Foz do Neiva, Rio de Moinhos, Esposende (breakwater), Ofir (southern breakwater), Pedrinhas, Apúlia, Santo André, Verde, Carvalhido, Póvoa de Varzim (marina), Caxinas, Forte de São João, Azurara (breakwater), Areia, Mindelo, Facho, Sampaio, Labruge, Angeiras (Maelas), Angeiras (Praia dos Barcos), Praia Central, Funtão, Pedras do Corgo, Pedras da Agudela, Memória, Cabo do Mundo, Boa Nova, Leça (Piscina das Marés), Leça (breakwater), Matosinhos (northern breakwater), Castelo do Queijo, Homem do Leme, Salgueiros, Valadares, Senhor da Pedra, Aguda, Bairro Piscatório (northern breakwater), Paramos (southern breakwater), Maceda (breakwater), Torreira (breakwater), Barra (northern breakwater), Barra (southern breakwater), Costa Nova (northern breakwater), Costa Nova (southern breakwater), Poço da Cruz (breakwater), Figueira da Foz and Buarcos. In central Portugal, the locations Figueira da Foz, Nazaré, São Martinho do Porto, Baleal and Papoa were surveyed. While most of these sites are natural rocky shores (Table 1), 17 are composed of artificial hard substrate amongst sandy beaches (Table 2). Some sites were surveyed twice.

For this work, we selected 34 intertidal macroalgae species to study possible changes in their abundance close to their geographic range limit (Suppl. material 1). We chose conspicuous macro-algae species as they are not only easy to observe and identify, but also less likely to be overlooked. This is important because reliable absence data is as essential as reliable presence data when assessing distribution change through time. The choice of species followed Lima et al. (2007) in order to obtain a comparable dataset. Briefly, we selected species that either have their absolute range limit or a distribution gap within or near the study area (Lima et al. 2007). We classified the species as warm-water, cold-water or neutral, based on how their Species Temperature Index (STI) compares with the median temperature of all surveyed sites in NW Iberia. STI was calculated using the median sea surface temperature (SST) of all coastal sites where the species is present in the North Atlantic Ocean, obtained by averaging 40 years of daily SST (1982–2021) from NOAA 1/4 arc-degree Daily Optimum Interpolation SST version 2, dOISST.v.2 (Banzon et al. 2016). This yielded a list of 19 species of cold-water affinity (macroalgae for which the species STI is lower than the median temperature in the study area), 10 species of warm-water affinity (macroalgae for which the STI is lower than the median temperature of the study area and five neutral species (with STI higher than the median temperature of some sites, but lower than others). From these, 30 are considered native species while four are invasive species.

### Sampling description

**Figure 3.**
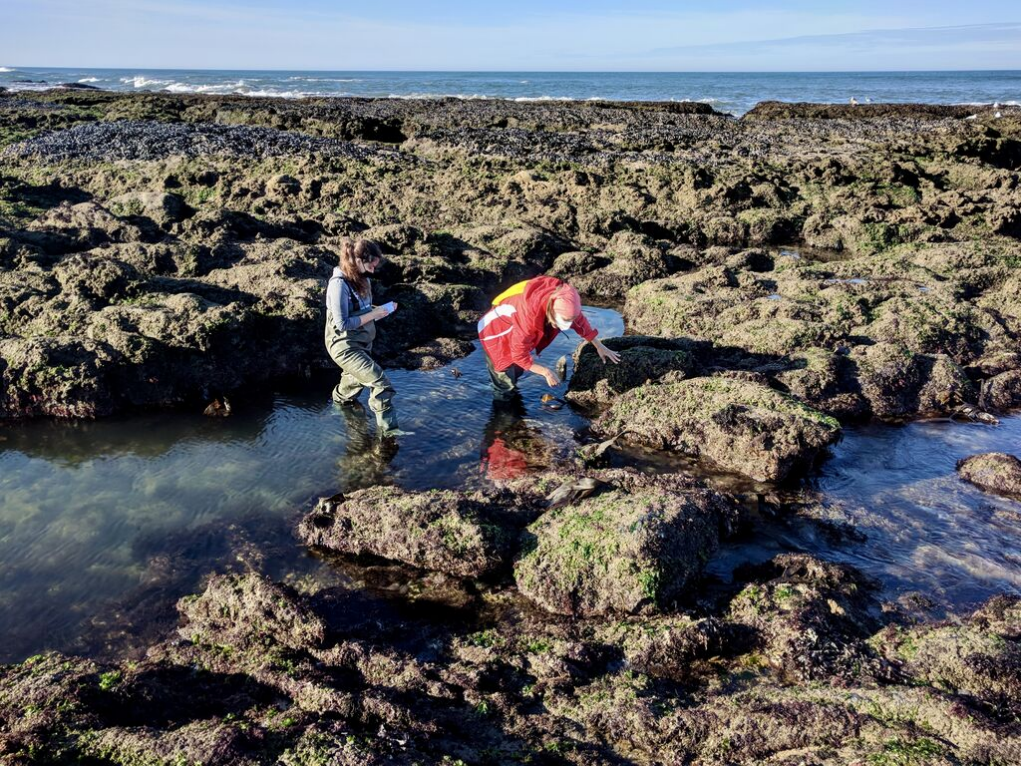
Two-person team recording SACFOR abundances at Aguda on 18/10/2020.

All locations were surveyed by a two-people team during spring low tides (the average low tide level was 1.52 +/- 0.14 m below mean sea water level, Fig. 3, Table 1, Table 2). Two locations were surveyed per day, one while the tide was still going out and another when the tide was already starting to rise. Each location was thoroughly surveyed for at least 60 minutes, except for most breakwaters which, owing to their relatively small area, were typically surveyed in less time. A semi-quantitative estimation of abundance was assigned to each selected species. We used a modified version of the scale established by Crisp and Southward (1958) — **SACFOR**, where abundances were encoded from 6 to 0 (where 6 means **S**uperabundant; 5, **A**bundant; 4, **C**ommon; 3, **F**requent; 2, **O**ccasional; 1, **R**are; and 0, not found).

**Figure 4.**
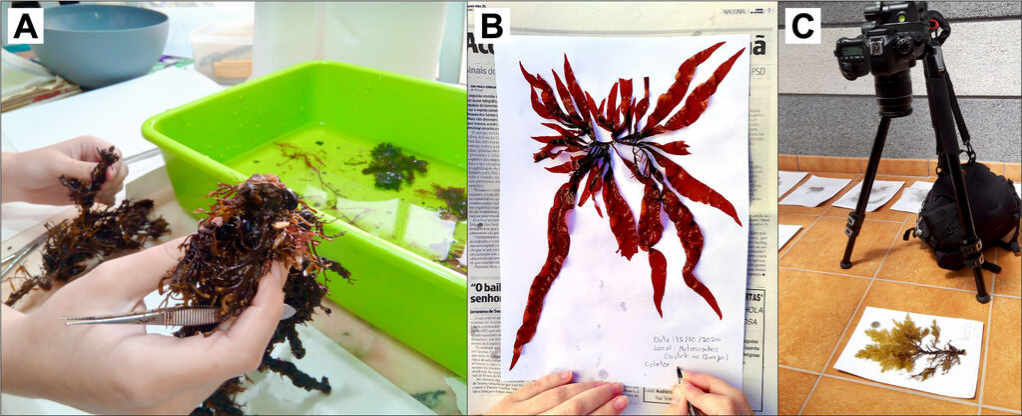
Representation of steps taken in the creation of herbarium: A - cleaning of specimens; B - identification of specimens, their collection location, date and collectors; C - photographing specimens.

Additionally, a herbarium was created with one specimen preserved per observed species per site, when feasible (Fig. 4). The algae collected were placed on drawing paper together with information on the species name, the location of collection, date and the sample collectors. Specimens were covered by non-adherent paper and placed between journal paper to dry. All specimens were piled and a weight was added on top so that the pressure helped to flatten them and stick them to the paper. Photos of each specimen were taken to duplicate the information in digital support. These photos were taken with a Canon EOS 6D camera fitted with a 28-80 mm objective at a fixed distance (55 cm), without zoom and with a €1 coin to serve as scale. Digital photos are available as an occurrence daset in GBIF (Pereira et al. 2021a), described below in Data Resources.

### Quality control

In addition to AlgaeBase (Guiry and Guiry 2021), authoritative identification guides and keys for the Eastern Atlantic and Mediterranean were used (Dixon and Irvine 1977, Hiscock 1979, Chapman and Goudey 1983, Irvine 1983, Hiscock 1986, Bárbara and Cremades 1987, Christensen 1987, Fletcher 1987, Burrows 1991, Cabioc'h et al. 1992, Maggs and Hommersand 1993, Irvine and Chamberlain 1994, Molenaar et al. 1996, Stuart et al. 1999, Brodie and Irvine 2003, Faes and Viejo 2003, Aziza et al. 2008, Araújo et al. 2009, Bárbara 2009, Vieira et al. 2010, Araújo et al. 2011, Edwards et al. 2012, Bárbara 2013, Bunker et al. 2017, Benita et al. 2018, Poza et al. 2019). All scientific names were standardised against the WoRMS - The World Register of Marine Species using the Taxon Match tool accessed on 07-07-2021 (WoRMS Editorial Board 2021).

### Step description

The steps that led to the final release of the dataset were as follows: (1) In-situ identification of species and attribution of a semi-quantitative abundance SACFOR score; (2) collection of specimens; (3) preservation of specimens in a herbarium; (4) photographing of each specimen as to duplicate the information in digital support; (5) standardisation of taxonomy against the World Register of Marine Species; (6) exporting of data as a DarwinCore Archive and (7) generation of dataset-level metadata.

## Geographic coverage

### Description

Sampling was done along three major rocky stretches of coast in north-western Iberia, covering the regions of Galicia, northern Portugal and central Portugal.

### Coordinates

39.37344 and 43.04422 Latitude; -8.64943 and -9.37772 Longitude.

## Taxonomic coverage

### Description

A total of 34 algae species were surveyed (19 Ochrophyta, 13 Rhodophyta and 2 Chlorophyta): *Ascophyllumnodosum*, *Asparagopsisarmata*, *Calliblepharisciliata*, *Chondruscrispus*, *Codiumadhaerens*, *Delesseriasanguinea*, *Desmarestialigulata*, *Dictyopterispolypodioides*, *Dilseacarnosa*, *Dumontiacontorta*, *Fucusserratus*, *Fucusspiralis*, *Gelidiumcorneum*, *Grateloupiaturuturu*, *Halidryssiliquosa*, *Halopithysincurva*, *Himanthaliaelongata*, *Hypneamusciformis*, *Laminariaochroleuca*, *Laminariahyperborea*, *Leathesiamarina*, *Padina pavonica*, *Palmariapalmata*, *Pelvetiacanaliculata*, *Petaloniafascia*, *Phycodrysrubens*, *Phyllophoracrispa*, *Saccharinalatissim*a, *Saccorhizapolyschides*, *Sargassumflavifolium*, *Sargassummuticum*, *Treptacanthabaccata*, *Undariapinnatifida* and *Valoniautricularis*. When it was not possible to discern between *L.ochroleuca* and *L.hyperborea* (at the juvenile stage), specimens were classified at the genus level, *Laminaria* sp. Full taxonomic description is presented on Suppl. material 1.

### Taxa included

**Table:** 

Rank	Scientific Name	
phylum	Ochrophyta	
phylum	Rhodophyta	
phylum	Chlorophyta	

## Temporal coverage

**Data range:** 2020-10-15 – 2021-7-26.

## Usage licence

### Usage licence

Open Data Commons Attribution License

### IP rights notes

Data users are free to share, create and adapt the dataset as long as they adequately attribute (cite) this work.

## Data resources

### Data package title

A survey of intertidal macroalgae species distribution along the north-western Iberian coast in 2020/2021 (occurrence/abundance/herbarium specimens)

### Resource link

http://ipt.gbif.pt/ipt/resource?r=2021_iberianpeninsula; https://doi.org/10.15468/9t2gxy

### Alternative identifiers

https://doi.org/10.15468/247z4g; http://ipt.gbif.pt/ipt/resource?r=herbarium

### Number of data sets

2

### Data set 1.

#### Data set name

Intertidal macroalgae species distribution along the north-western Iberian coast in 2020/2021

#### Data format

Darwin Core archive

#### Number of columns

37

#### Download URL

https://www.gbif.org/dataset/c1e31227-6595-4797-b75a-d9d9f75e4cca

#### Description

The data presented in this paper results from visual surveys done along the north-western Iberian rocky intertidal in 2020 and 2021, focusing on 34 macroalgae species. The dataset published in GBIF has the structure of a Sampling event dataset with two data subsets: Events (Core) and Associated occurrences. These data have been published (Pereira et al. 2021b) as a Darwin Core Archive (DwCA), which is a standardised format for sharing biodiversity data (Wieczorek et al. 2012). The Sampling Event (Core) contains 77 records (eventID). The extension data (Associated Occurrences) sheet has 2632 occurrences.

**Data set 1.** 

Column label	Column description
eventID	Unique identifier associated with an event.
samplingProtocol	Sampling method used during the event.
samplingEffort	Description of effort during the sampling event.
eventDate	The date of the event.
year	The year of the event.
month	The month of the event.
day	The day of the event.
country	Country where the event took place.
countryCode	The unique code of the country where the event took place.
Locality	A description commonly associated with the sampling place.
locationID	An identifier for the location information from Geonames.
decimalLatitude	The geographical latitude of the event.
decimalLongitude	The geographical longitude of the event.
geodeticDatum	The geodetic datum upon which the geographical coordinates are based.
coordinatePrecision	The precision of the coordinates.
coordinateUncertaintyInMetres	The uncertainty of the coordinates, in metres.
type	Type of dataset.
ownerInstitutionCode	Identifier code of the owner institution.
habitat	The habitat in which the event took place.
waterBody	The water body in which the event took place.
rightsHolder	The rights holder of the dataset.
occurrenceID	Unique identifier associated with the occurrence of a species.
basisOfRecord	The specific nature of the data record.
organismQuantity	An enumeration value for the quantity of a species.
organismQuantityType	The quantification scale of the quantity of a species.
occurrenceStatus	A statement about the presence or absence of a species in a location.
scientificName	The full scientific name, with authorship and date information, if known.
scientificNameID	Unique identifier of a species, obtained from WoRMS.
kingdom	The full scientific name of the kingdom in which the taxon is classified.
phylum	The full scientific name of the phylum in which the taxon is classified.
class	The full scientific name of the class in which the taxon is classified.
order	The full scientific name of the order in which the taxon is classified.
family	The full scientific name of the family in which the taxon is classified.
genus	The full scientific name of the genus in which the taxon is classified.
specificEpithet	The specific epithet of the species.
taxonRank	The taxonomic rank of the most specific name in scientificName.
recordedBy	People responsible for sampling the occurrence.

### Data set 2.

#### Data set name

Herbarium collection of intertidal macroalgae biodiversity along the north-western Iberian coast in 2020/2021

#### Number of columns

29

#### Download URL

https://www.gbif.org/dataset/e9543008-b26d-458e-b334-a201c5c3b7e5

#### Description

This collection comprises 378 herbarium specimens preserved, dried and stored at CIBIO and 378 photos of these specimens stored digitally (Pereira et al. 2021a). These herbarium specimens were collected while surveying the occurrence and abundance of macroalgae species in north and central Portugal and northwest Spain.

**Data set 2.** 

Column label	Column description
occurrenceID	Unique identifier associated with an occurrence.
basisofRecord	The specific nature of the data record.
eventDate	The date when the occurrence was observed.
year	The year when the occurrence was observed.
month	The month when the occurrence was observed.
day	The day when the occurrence was observed.
scientificName	The full scientific name, with authorship and date information, if known.
kingdom	The full scientific name of the kingdom in which the taxon is classified.
phylum	The full scientific name of the phylum in which the taxon is classified.
class	The full scientific name of the class in which the taxon is classified.
order	The full scientific name of the order in which the taxon is classified.
family	The full scientific name of the family in which the taxon is classified.
genus	The full scientific name of the genus in which the taxon is classified.
specificEpithet	The specific epithet of the species.
taxonRank	The taxonomic rank of the most specific name in scientificName.
decimalLatitude	The geographical latitude where the occurrence was recorded.
decimalLongitude	The geographical longitude where the occurrence was recorded.
geodeticDatum	The geodetic datum upon which the geographical coordinates are based.
coordinateUncertaintyInMeters	The uncertainty of the coordinates, in metres.
continent	The continent where the occurrence was recorded.
country	The country where the occurrence was recorded.
countryCode	The standard code for the country where the occurrence was recorded.
type	The nature or genre of the resource.
associatedMedia	A list (concatenated and separated) of identifiers (URI) of media associated with the Occurrence.
institutionCode	The name (or acronym) in use by the institution having custody of the object(s) or information referred to in the record.
recordedBy	People responsible for sampling the occurrence.
establishmentMeans	Statement about whether an organism or organisms have been introduced to a given place and time through the direct or indirect activity of modern humans.
preparations	A list (concatenated and separated) of preparations and preservation methods for a specimen.
rightsHolder	An organisation owning or managing rights over the resource.

## Additional information

A total of 34 macroalgae species were surveyed: 19 Ochrophyta, 13 Rhodophyta and two Chlorophyta (Pereira et al. 2021b). More taxonomic information per species is presented in Suppl. material 1.

## Supplementary Material

F592F307-6344-5748-B052-40B1A6CB79E0 10.3897/BDJ.10.e80798.suppl1 Supplementary material 1 Taxonomic and biogeographic information Data type Taxonomic and biogeographic data Brief description List of species surveyed, scientific name ID from the World Register of Marine Species (WoRMS) and taxonomic ranks. File: oo_635548.txt https://binary.pensoft.net/file/635548 Joana Pereira, Cátia Monteiro, Rui Seabra and Fernando P. Lima

## Figures and Tables

**Table 1.** Natural rocky shore sites surveyed, their coordinates and date of the survey. The coordinates were obtained from GoogleMaps imagery. Locations are listed from north to south.

**ID**	**Location**	**Latitude**	**Longitude**	**Date**	**Astronomical low tide height (m below mean sea water level)**
1	Cabo Touriñán	43.04423	-9.28810	24/05/2021	-1.52
2	Corveiro	42.90442	-9.26077	26/05/2021	-1.68
3	Quenxe	42.93650	-9.18958	26/05/2021	-1.68
4	Ximprón	42.79679	-9.14016	25/05/2021	-1.63
5	Punta Outeiriño	42.74564	-9.07681	25/05/2021	-1.63
6	Corrubedo	42.57665	-9.08985	26/06/2021	-1.45
7	O Touro	42.54606	-8.98397	26/06/2021	-1.45
8	Prado	42.15921	-8.81940	27/05/2021	-1.62
9	Faro Vello de Silleiro	42.11185	-8.89945	27/05/2021	-1.62
10	Oia	42.00199	-8.87770	28/05/2021	-1.49
11	Fedorento	41.91017	-8.87801	28/05/2021	-1.48
12	Moledo	41.83815	-8.87491	19/10/2020	-1.69
12	Moledo	41.83908	-8.87529	25/06/2021	-1.43
13	Vila Praia de Âncora	41.81940	-8.87205	17/12/2020	-1.45
14	Afife	41.78439	-8.87168	17/12/2020	-1.69
14	Afife	41.78072	-8.87014	19/10/2020	-1.45
15	Montedor	41.74292	-8.87591	29/01/2021	-1.37
16	Forte da Vigia	41.69959	-8.85507	16/11/2020	-1.72
17	Praia Norte	41.69983	-8.85472	16/11/2020	-1.72
19	Amorosa	41.64290	-8.82338	12/01/2021	-1.39
20	Foz do Neiva	41.61095	-8.80893	16/12/2020	-1.55
21	Rio de Moinhos	41.57362	-8.79846	16/12/2020	-1.55
25	Apúlia	41.48267	-8.77886	17/11/2020	-1.62
26	Santo André	41.41663	-8.78827	15/01/2021	-1.42
27	Verde	41.38542	-8.77433	15/01/2021	-1.42
28	Carvalhido	41.38149	-8.77150	30/03/2021	-1.79
30	Caxinas	41.36220	-8.76045	13/01/2021	-1.46
31	Forte de São João	41.34108	-8.75073	13/01/2021	-1.46
33	Areia	41.33355	-8.73993	14/01/2021	-1.49
34	Mindelo	41.31052	-8.74136	14/01/2021	-1.49
35	Facho	41.29241	-8.73419	15/12/2020	-1.58
36	Sampaio	41.27956	-8.72914	15/12/2020	-1.58
37	Labruge	41.27309	-8.72900	16/01/2021	-1.32
38	Angeiras (Maelas)	41.26615	-8.72829	31/03/2021	-1.68
39	Angeiras (Praia dos Barcos)	41.26510	-8.72818	16/01/2021	-1.32
40	Praia Central	41.26187	-8.72686	31/01/2021	-1.48
41	Funtão	41.26041	-8.72494	15/11/2020	-1.66
42	Pedras do Corgo	41.24931	-8.72591	15/11/2020	-1.66
43	Pedras da Agudela	41.24163	-8.72795	14/11/2020	-1.55
44	Memória	41.23528	-8.72433	17/10/2020	-1.71
45	Cabo do Mundo	41.22115	-8.71577	17/10/2020	-1.71
46	Boa Nova	41.20458	-8.71553	16/10/2020	-1.59
47	Leça (Piscina das Marés)	41.19231	-8.70742	16/10/2020	-1.59
50	Castelo do Queijo	41.16746	-8.69016	15/10/2020	-1.38
50	Castelo do Queijo	41.16722	-8.69020	23/06/2021	-1.35
51	Homem do Leme	41.15903	-8.68538	14/12/2020	-1.54
51	Homem do Leme	41.15903	-8.68538	14/02/2021	-1.37
52	Salgueiros	41.12148	-8.66652	18/11/2020	-1.45
53	Valadares	41.08964	-8.65700	18/11/2020	-1.46
54	Senhor da Pedra	41.06894	-8.65836	18/10/2020	-1.76
54	Senhor da Pedra	41.06846	-8.65848	24/06/2021	-1.40
55	Aguda	41.04554	-8.65282	18/10/2020	-1.76
55	Aguda	41.04613	-8.65325	24/06/2021	-1.40
64	Buarcos	40.17751	-8.90354	03/03/2021	-1.46
65	Pedrogão	39.91612	-8.95537	12/04/2021	-1.31
67	Nazaré	39.60384	-9.08041	01/03/2021	-1.63
68	São Martinho do Porto	39.51151	-9.14207	26/07/2021	-1.28
69	Papôa	39.37344	-9.37773	02/03/2021	-1.58
70	Baleal	39.37586	-9.33981	02/03/2021	-1.58

**Table 2.** Artificial substrate locations surveyed and their correspondent coordinates and date of the survey. The coordinates were obtained from GoogleMaps imagery. Locations are listed from north to south.

**ID**	**Location**	**Latitude**	**Longitude**	**Date**	**Astronomical low tide height (m below mean sea water level)**
18	Cabedelo (breakwater)	41.67923	-9.83669	15/03/2021	-1.35
22	Esposende (breakwater)	41.54149	-8.79361	28/03/2021	-1.65
23	Ofir (southern breakwater)	41.51551	-8.78768	28/03/2021	-1.65
24	Pedrinhas	41.50590	-8.78829	17/11/2020	-1.62
29	Póvoa de Varzim (marina)	41.37615	-8.76433	28/03/2021	-1.65
32	Azurara (breakwater)	41.33919	-8.74709	30/03/2021	-1.79
48	Leça (breakwater)	41.18622	-8.70760	31/03/2021	-1.68
49	Matosinhos (northern breakwater)	41.17800	-8.69908	23/06/2021	-1.35
56	Bairro Piscatório (northern breakwater)	40.99809	-8.64944	12/03/2021	-1.37
57	Paramos (southern breakwater)	40.97362	-8.65141	12/03/2021	-1.37
58	Maceda (breakwater)	40.93121	-8.66139	12/03/2021	-1.37
59	Barra (northern breakwater)	40.64102	-8.75212	14/03/2021	-1.44
60	Barra (southern breakwater)	40.62783	-8.75108	14/03/2021	-1.44
61	Costa Nova (northern breakwater)	40.61955	-8.75398	14/03/2021	-1.44
62	Costa Nova (southern breakwater)	40.61949	-8.74819	14/04/2021	-1.24
63	Poço da Cruz (breakwater)	40.48955	-8.79457	13/03/2021	-1.43
66	Figueira da Foz (marina)	40.14684	-8.86726	13/03/2021	-1.41
